# Left atrial geometry in an ovine ischemic mitral regurgitation model: implications for transcatheter mitral valve replacement devices with a left atrial anchoring mechanism

**DOI:** 10.1186/s13019-021-01654-0

**Published:** 2021-10-10

**Authors:** Akito Imai, Arash Khamooshian, Keitaro Okamoto, Yoshiaki Saito, Inez J. Wijdh-den Hamer, Massimo A. Mariani, Matthew J. Gillespie, Robert C. Gorman, Joseph H. Gorman, Wobbe Bouma

**Affiliations:** 1 Gorman Cardiovascular Research Group, University of Pennsylvania, Philadelphia, PA USA; 2 Department of Cardiovascular Surgery, University of Tsukuba, Tsukuba, Ibaraki Japan; 3 Department of Cardiothoracic Surgery, University Medical Center Groningen, University of Groningen, Hanzeplein 1, P.O. Box 30001, 9700 RB Groningen, The Netherlands; 4 Department of Cardiovascular Surgery, Oita University, Oita, Japan; 5 Department of Thoracic and Cardiovascular Surgery, Hirosaki University School of Medicine, Hirosaki, Japan; 6 Department of Pediatrics, Children’s Hospital of Philadelphia, University of Pennsylvania, Philadelphia, PA USA; 7 Department of Surgery, University of Pennsylvania, Philadelphia, PA USA; 8 Onocor LLC, Philadelphia, PA USA

**Keywords:** Ischemic mitral regurgitation (IMR), Left atrial anchoring, Magnetic resonance imaging (MRI), (Transcatheter) mitral valve replacement ((T)MVR)

## Abstract

**Background:**

Transcatheter mitral valve replacement (TMVR) is a challenging, but promising minimally invasive treatment option for patients with mitral valve disease. Depending on the anchoring mechanism, complications such as mitral leaflet or chordal disruption, aortic valve disruption or left ventricular outflow tract obstruction may occur. Supra-annular devices only anchor at the left atrial (LA) level with a low risk of these complications. For development of transcatheter valves based on LA anchoring, animal feasibility studies are required. In this study we sought to describe LA systolic and diastolic geometry in an ovine ischemic mitral regurgitation (IMR) model using magnetic resonance imaging (MRI) and echocardiography in order to facilitate future research focusing on TMVR device development for (I)MR with LA anchoring mechanisms.

**Methods:**

A group of 10 adult male Dorsett sheep underwent a left lateral thoracotomy. Posterolateral myocardial infarction was created by ligation of the left circumflex coronary artery, the obtuse marginal and diagonal branches. MRI and echocardiography were performed at baseline and 8 weeks after myocardial infarction (MI).

**Results:**

Six animals survived to 8 weeks follow-up. All animals had grade 2 + or higher IMR 8 weeks post-MI. All LA geometric parameters did not change significantly 8 weeks post-MI compared to baseline. Diastolic and systolic interpapillary muscle distance increased significantly 8 weeks post-MI.

**Conclusions:**

Systolic and diastolic LA geometry do not change significantly in the presence of grade 2 + or higher IMR 8 weeks post-MI. These findings help facilitate future tailored TMVR device development with LA anchoring mechanisms.

## Background

Conventional mitral valve surgery (either repair or replacement) is the standard of care for patients with severe symptomatic mitral valve (MV) regurgitation or stenosis. Although a substantial portion of these patients are not eligible for surgery because of comorbidity and a high surgical risk [1]. Transcatheter mitral valve replacement (TMVR) and repair (TMVr) are enticing, but slowly evolving therapeutic options for patients with MV disease, in particular for those with a high surgical risk and those not eligible for an open procedure. Edge-to-edge leaflet repair with the MitraClip (Abbott, Chicago, IL) is the only TMVr system with the most experience to date. However, not all MV pathologies are suitable for this technique and residual moderate to severe mitral regurgitation (MR) has been reported in approximately 10% of the patients [2, 3]. For the treatment of severe MR, TMVR has emerged as an alternative. Although transcatheter aortic valve replacement (TAVR) is a well-established treatment option for patients with severe aortic stenosis, the experience with TMVR remains limited [4]. Some experience has been gained with the off-label use of TAVR devices for “valve-in-ring” remedial procedures for failed mitral valve repair [5–7], but the development of dedicated TMVR valves is currently still in its infancy [8–10]. The complexity of MV anatomy, its proximity to the left ventricular outflow tract (LVOT) and the hemodynamic forces a TMVR device must anchor and function under, have generated significant design challenges. Potential complications of TMVR are LVOT obstruction, device embolization, left ventricular dysfunction (e.g. chordae tendineae rupture) and death [11]**.** Despite these technical, anatomical, and functional constraints, substantial progress has been made over the last several years. The majority of TMVR devices currently being developed use a varying combination of annular interference fit, leaflet grasping or apical fixation as anchoring strategies. Left atrial anchoring strategies have been proposed, but less well explored.

The recently introduced supra-annular AltaValve (4C Medical Technologies, Brooklyn Park, Minnesota) is based on a LA anchoring mechanism and directly addresses MR without replacing the native mitral valve or disrupting the mitral annulus and leaflets, subvalvular apparatus, LVOT or aortic valve [12].

Though relatively unexplored the LA anchoring mechanism may prove a very efficient design option for development of TMVR devices. Further development of this anchoring strategy will require feasibility and efficacy studies in normal and ischemic MR (IMR) animal models. To carry out these studies animal prototypes will need to be created based on LA size and geometry. This information is not currently available in the literature. Therefore, we sought to describe LA systolic and diastolic geometry in both normal sheep and an ovine IMR model using magnetic resonance imaging (MRI) and echocardiography. This data will facilitate future research focusing on TMVR device development for (I)MR with LA anchoring mechanisms.

## Methods

### Surgical protocol

The study protocol was reviewed and approved by the University of Pennsylvania’s Institutional Animal Care and Use Committee (IACUC) and was in compliance with the “Guide for the Care and Use of Laboratory Animals” compliance with (US National Institutes of Health Publication No. 85-23, revised 1996).

Ten adult male Dorsett sheep (49.2 + / − 2.6 kg) were sedated with intramuscular Ketamine injections (25–30 mg/kg), intubated, and mechanically ventilated. General anesthesia was maintained on mixed Isoflurane (1.5–3.0%) and oxygen, which was delivered by volume-controlled ventilation (tidal volume 10–15 mg/kg). The surface electrocardiogram and arterial blood pressure were monitored. All animals underwent a left lateral thoracotomy and partial removal of the fifth rib to allow ligation of the left circumflex artery, obtuse marginal branches and diagonal branches to create a posterolateral myocardial infarction (MI). After hemodynamic and electrophysiological stabilization, the thoracotomy was closed, and the animal was permitted to recover.

MRI and echocardiography were performed in all animals at baseline and 8 weeks post-MI. Six animals survived to 8 weeks follow-up. All six animals had grade 2+ or higher IMR 8 weeks post-MI.

### MRI image acquisition

MRI was performed with a 3.0 Tesla Siemens Tim Trio (Siemens, München, Germany). Imaging was performed with the use of a body matrix surface coil and a spine phased array coil. Imaging was acquired using both retrospective cardiac gating and breath-holding. Cardiac gating was performed by placement of a pressure catheter (Millar Instruments, Inc. Houston, TX) in the left ventricle (LV). The imaging protocol for sizing of the left atrium, left ventricle, and valvular landmarks and structures was as follows:

Short axis TruFisp cines were acquired in two dimensions of the entire LV with the following parameters: slice thickness—4 mm, FOV—280 mm × 166 mm, in-plane resolution 1.1 mm × 1.1 mm, TR—27.52 ms, TE—1.46 ms, Flip angle—44.

Single slice GRE cines of the following views were acquired: 4 chamber, 2 chamber, vertical long-axis. These views were obtained with the following parameters: slice thickness—4 mm, FOV—300 mm × 206 mm, in-plane resolution 1.6 mm × 1.6 mm, TR—28.4 ms, TE—2.85 ms, Flip angle—12.

Single slice phase contrast through-plane at mitral annulus for IMR evaluation was obtained with the following parameters: slice thickness—4 mm, FOV—300 mm × 206 mm, in-plane resolution 2.1 × 1.6, TR—40.85 ms, TE—3.33 ms, Flip angle—12, VENC range 75 cm/s.

All MRI images were suitable for analysis. Reconstructed images were transferred to an external workstation and subsequently analyzed.

### MRI image analysis

**Fig. 1:**
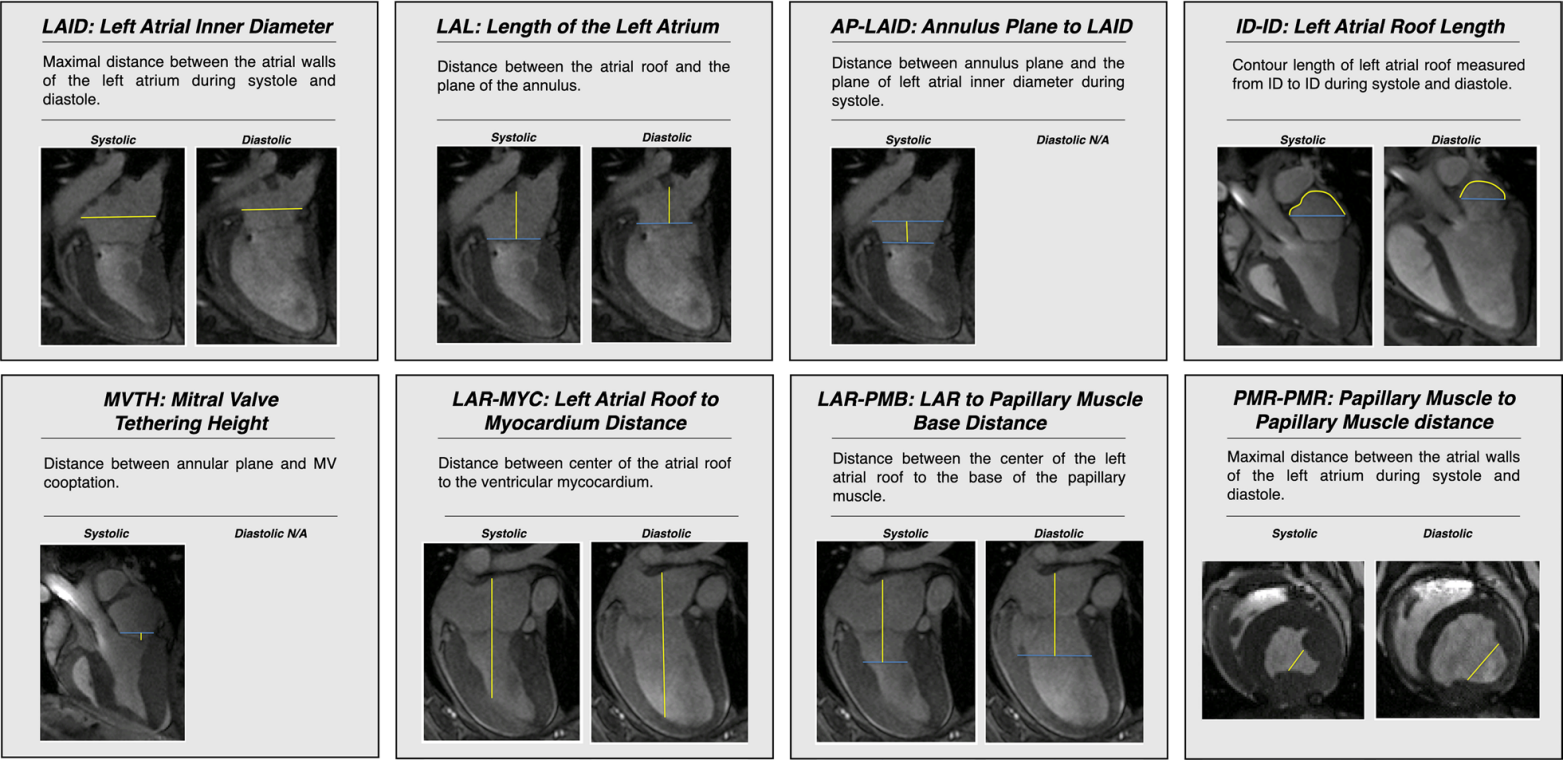
MRI parameters measured at baseline and 8 weeks post myocardial infarction

The following parameters were measured by MRI at baseline and 8 weeks after myocardial infarction: (*1) left atrial inner diameter (LAID), (2) length of the left atrium (LAL)*, (3) *annulus plane to LAID (AP-LAID), (4) left atrial roof length (ID-ID), (5) mitral valve tethering height (MVTH), (6) left atrial roof to myocardium distance (LAR-MYC), (7*) *LAR to papillary muscle base distance (LAR-PMB), (8) papillary muscle to papillary muscle distance (PMR-PMR)* (Fig. 1)*.*

### Statistics

Continuous variables were expressed as mean ± standard deviation (SD). Comparisons within groups were performed using the paired samples *t*-test (two-sided) as appropriate for continuous variables. All calculations were performed using SPSS Statistics (IBM SPSS Statistics 22.0; IBM Corporation, Chicago, IL). Statistically significant differences were established at *P* < 0.05.

## Results

### Geometric MRI data

Ten animals were included in this study at baseline, 6 animals were alive 8 weeks post-MI and underwent follow-up MRI. The geometric MRI data are shown in Table 1. Eight weeks post-MI interpapillary muscle distance enlarged significantly both in systole and diastole. Grade 2+ or more IMR was observed after 8 weeks in all cases. All LA geometric parameters did not change significantly 8 weeks post-MI compared to baseline.

**Table 1:** Geometric ovine MRI data at baseline and 8 weeks post myocardial infarction (systolic and diastolic)

Variable^a^	Systolic			Diastolic		
	Baseline	8 weeks post-MI	*P* value	Baseline	8 weeks post-MI	*P* value
Let atrial inner diameter (LAID), mm	55.9 ± 6.9	53.8 ± 4.8	0.580	44.7 ± 3.5	47.0 ± 6.6	0.566
Left atrial length (LAL), mm	33.2 ± 4.0	33.7 ± 2.8	0.847	26.6 ± 3.6	31.0 ± 2.3	0.091
Annulus plane to LAID, mm	18.5 ± 2.7	19.5 ± 1.9	0.381	−	−	−
Left atrial roof contour, mm	61.4 ± 5.0	62.2 ± 2.3	0.754	46.7 ± 6.7	52.8 ± 5.6	0.165
Mitral valve height, mm	4.6 ± 0.9	4.6 ± 1.0	0.961	−	−	−
Left atrial roof to myocardium, mm	77.7 ± 4.5	80.8 ± 5.0	0.276	92.1 ± 4.2	88.5 ± 5.0	0.201
Left atrial roof to papillary muscle base, mm	55.8 ± 4.6	58.0 ± 2.9	0.423	49.9 ± 5.3	56.8 ± 3.1	0.057
Interpapillary muscle distance, mm	16.8 ± 4.5	28.5 ± 6.1	< 0.001	29.3 ± 4.8	36.9 ± 3.7	0.004

LAID = left atrial inner diameter; LAL = left atrial length; MI = myocardial infarction; MRI = magnetic resonance imaging

^a^Data are presented as mean ± SD

## Discussion

TMVR and TMVr are new therapeutic options for patients with mitral valve disease. Especially TMVR has emerged as an alternative to surgery for patients with an increased operative risk [2]. However, TMVR still faces many technical and anatomical challenges [11]. A variety of TMVR designs are currently under investigation. TMVR design has to take into account the proximity of the MV to the LVOT, the complexity of the MV itself and hemodynamic forces. TMVR devices should not obstruct the LVOT, fit the MV annulus well, not cause left ventricular dysfunction by disrupting the chords and be anti-thrombogenic. The main challenge is designing a robust anchoring mechanism which meets these standards. Different designs have been introduced, ranging from annular interference fit, leaflet grasping, apical fixation to LA anchoring strategies [13].

Our group has explored several leaflet, annular and left ventricular TMVR anchoring strategies and have found that our previously published normal and IMR ovine LV and annular data [14–16] to be extremely helpful in designing prototypes for testing in animal models. We have not previously studied ovine LA anatomy and the literature on ovine LA anatomy is very sparse. To date, only one device has been proposed with a LA anchoring mechanism; the supra-annular AltaValve (4C Medical Technologies, Brooklyn Park, MN) [12]. We believe LA anchoring is a potentially effective strategy and undertook the current study to provide geometric baseline and post-infarct ovine LA data to facilitate its development.

Benefits of a LA anchoring design include the lack of need to replace the native mitral valve or disrupt the mitral annulus and leaflets, subvalvular apparatus, LVOT and aortic valve. In this study we provide baseline and 8 weeks post-infarct data regarding ovine LA geometry. This study shows that LA geometry remains relatively stable in the presence of grade 2+ or more IMR in the first 8 weeks post-MI. The specific implications of these findings are that initial development and testing of TMVR devices with a LA anchoring mechanism (such as a LA nitinol stent) can be done in normal sheep. This is an important finding, because it eliminates the additional time and expense required to produce the ovine IMR model. Once a design has been shown to be effective in normal sheep, subsequent testing can then be conducted more efficiently in our ovine chronic IMR models.

Device testing in long-term IMR ovine models are a next step in moving towards human testing. While this study shows that LA geometry remains relatively stable in the presence of grade 2+ or more IMR in the first 8 weeks post-MI, additional studies are required to explore what happens after a longer timeframe in this ovine IMR model.

It should be stressed that the current data is only helpful in developing animal prototypes for proof of principle studies. Devices for human use, while being similarly designed, will have to be re-scaled based on human LA geometrical data. Our group is currently analyzing our large library of human IMR echocardiography studies to generate this information.

## Limitations

The number of animals in this study is relatively small. Although we used a chronic post-MI model, additional LA geometrical and functional changes are expected to occur over a long period of time in the presence of IMR. This may have implications for LA anchoring TMVR device development and is currently being studied. IMR was measured semiquantitatively with jet area/LA area. Alternative validated methods for quantitative IMR severity assessment, including regurgitant volume and effective regurgitant orifice, were not available in this study.

## Conclusions

Systolic and diastolic LA geometry do not change significantly in the presence of grade 2+ or higher IMR 8 weeks post-MI in an ovine model. These findings help facilitate future tailored TMVR device development with LA anchoring mechanisms.

## Data Availability

The datasets used and/or analysed during the current study are available from the corresponding author on reasonable request.

## Abbreviations

(AP-)LAID: (Annulus plane to) left atrial inner diameter
IACUC: Institutional animal care and use committee
ID-ID: Left atrial roof length
(I)MR: (Ischemic) mitral regurgitation
LA(L): Left atrial (length)
LAR-MYC: Left atrial roof to myocardium distance
LAR-PMB: Left atrial roof to papillary muscle base
LV: Left ventricle
LVOT: Left ventricular outflow tract
MI: Myocardial infarction
MRI: Magnetic resonance imaging
MV(TH): Mitral valve (tethering height)
PMR-PMR: Papillary muscle to papillary muscle distance
SD: Standard deviation
TAVR: Transcatheter aortic valve replacement
(T)MVr: (Transcatheter) mitral valve repair
(T)MVR: (Transcatheter) mitral valve replacement
